# Restoring myocardial infarction-induced long-term memory impairment by targeting the cystic fibrosis transmembrane regulator

**DOI:** 10.1016/j.ebiom.2022.104384

**Published:** 2022-11-30

**Authors:** Lotte Vanherle, Darcy Lidington, Franziska E. Uhl, Saskia Steiner, Stefania Vassallo, Cecilia Skoug, Joao M.N. Duarte, Sangeetha Ramu, Lena Uller, Jean-François Desjardins, Kim A. Connelly, Steffen-Sebastian Bolz, Anja Meissner

**Affiliations:** a Department of Experimental Medical Science, Lund University, Lund, Sweden; b Wallenberg Centre for Molecular Medicine, Lund University, Lund, Sweden; c Department of Physiology, University of Toronto, Toronto, Canada; d Keenan Research Centre for Biomedical Science, St. Michael's Hospital; Toronto, Ontario, Canada; e Department of Physiology, Institute of Theoretical Medicine, Medical Faculty, University of Augsburg, Augsburg, Germany; f German Centre for Neurodegenerative Diseases, Bonn, Germany

**Keywords:** Neurodegeneration, Cognitive impairment, Myocardial infarction, Microglia activation, Cystic fibrosis transmembrane conductance regulator, CFTR, cystic fibrosis transmembrane regulator, MI, myocardial infarction, Lum, lumacaftor, Iva, ivacaftor, PSD-95, post‑synaptic density protein 95, LCM, lipopolysaccharide-stimulated microglia conditioned medium, HF, heart failure, CBF, cerebral blood flow, LAD, left anterior descending coronary artery, EF, ejection fraction, NOR, Novel Object Recognition, IL, interleukin, Iba-1, ionised calcium-binding adapter molecule-1, CM, conditioned medium, WT, wild-type, CA1, cornu ammonis 1, DG, dentate gyrus

## Abstract

**Background:**

Cognitive impairment is a serious comorbidity in heart failure patients, but effective therapies are lacking. We investigated the mechanisms that alter hippocampal neurons following myocardial infarction (MI).

**Methods:**

MI was induced in male C57Bl/6 mice by left anterior descending coronary artery ligation. We utilised standard procedures to measure cystic fibrosis transmembrane regulator (CFTR) protein levels, inflammatory mediator expression, neuronal structure, and hippocampal memory. Using *in vitro* and *in vivo* approaches, we assessed the role of neuroinflammation in hippocampal neuron degradation and the therapeutic potential of CFTR correction as an intervention.

**Findings:**

Hippocampal dendrite length and spine density are reduced after MI, effects that associate with decreased neuronal CFTR expression and concomitant microglia activation and inflammatory cytokine expression. Conditioned medium from lipopolysaccharide-stimulated microglia (LCM) reduces neuronal cell CFTR protein expression and the mRNA expression of the synaptic regulator post-synaptic density protein 95 (PSD-95) *in vitro*. Blocking CFTR activity also down-regulates PSD-95 in neurons, indicating a relationship between CFTR expression and neuronal health. Pharmacologically correcting CFTR expression *in vitro* rescues the LCM-mediated down-regulation of PSD-95. *In vivo*, pharmacologically increasing hippocampal neuron CFTR expression improves MI-associated alterations in neuronal arborisation, spine density, and memory function, with a wide therapeutic time window.

**Interpretation:**

Our results indicate that CFTR therapeutics improve inflammation-induced alterations in hippocampal neuronal structure and attenuate memory dysfunction following MI.

**Funding:**

10.13039/501100004063Knut and Alice Wallenberg Foundation [F 2015/2112]; 10.13039/501100004359Swedish Research Council [VR; 2017-01243]; the 10.13039/501100001659German Research Foundation [DFG; ME 4667/2-1]; 10.13039/501100003792Hjärnfonden [FO2021-0112]; 10.13039/100018740The Crafoord Foundation; 10.13039/100007435Åke Wibergs Stiftelse [M19-0380], NMMP 2021 [V2021-2102]; the 10.13039/501100006189Albert Påhlsson Research Foundation; 10.13039/501100001728STINT [MG19-8469], 10.13039/501100003252Lund University; 10.13039/501100000024Canadian Institutes of Health Research [PJT-153269] and a 10.13039/100004411Heart and Stroke Foundation of Ontario Mid-Career Investigator Award.

**Research in context**

**Evidence before this study**

Heart failure frequently associates with cognitive decline with yet no treatment options. Cerebral cystic fibrosis transmembrane regulator (CFTR) is down-regulated in the brain after myocardial infarction.

**Added value of this study**

Neuroinflammation appears to link myocardial infarction to neuronal CFTR down-regulation. Inhibiting CFTR activity renders neuronal cells more susceptible to injury. Restoring CFTR expression normalises neuronal injury induced by myocardial infarction. The therapeutic time window for reversing structural and functional changes occurring in the hippocampus after myocardial infarction is large.

**Implications of all the available evidence**

Repurposing clinically available CFTR correctors may attenuate brain injury and improve cognitive function in patients with heart failure.

## Introduction

Approximately 15.9 million cases of myocardial infarction (MI) occur annually^1^ and currently, over 64 million people live with heart failure (HF) worldwide.^2^ Although improved acute cardiac care has increased MI survival, HF-related health care expenditures continue to rise due to the high incidence of cognitive impairment within the HF patient population.^3^, ^4^, ^5^ Cognitive impairment imposes a detrimental health burden on HF patients, as it negatively impacts quality of life, self-care management, treatment adherence, hospitalisation rates, and mortality.^6^ Interventions that prevent, slow down, or reverse cognitive decline in HF patients are desperately needed; however, a lack of mechanistic insight has limited the number of safe and effective pharmaceutical interventions.

There is a well-established relationship between hippocampal atrophy and cognitive decline.^7^^,^^8^ Thus, the hippocampus is a major target for research on heart–brain interactions that underlie cognitive decline in HF.^9^ Indeed, hippocampal atrophy, cognitive decline,^10^, ^11^, ^12^ and lower memory recall performance^13^^,^^14^ all associate with left ventricular dysfunction.^15^, ^16^, ^17^ The specific mechanisms that drive the MI-induced loss of hippocampal neurons are not known, but they are postulated to be involved in inflammatory processes within the brain,^18^^,^^19^. In this regard, microglia activation is thought to be a critical mediator of behavioural and cognitive changes following MI.^20^, ^21^, ^22^

Experimental mouse models of MI emulate several key features of brain alterations reported in patients, including reduced cerebral blood flow (CBF), changes in neuronal structure, and compromised neurological function.^23^^,^^24^ Our previous work demonstrated that experimental HF drives cortical neuron atrophy and cortex-dependent short-term memory impairment.^25^^,^^26^ Mechanistically, we correlated these alterations to impaired vascular reactivity caused by the down-regulation of the cystic fibrosis transmembrane regulator (CFTR) within the vascular wall.^25^ In addition to vascular cells, CFTR is also widely expressed in neuronal cells, both in rodents and in humans.^27^, ^28^, ^29^, ^30^, ^31^ This raises the intriguing prospect that therapeutically manipulating CFTR expression with clinically approved CFTR corrector compounds could have direct beneficial effects on neuronal health, in addition to improving cerebral perfusion.^25^ Based on our previous work,^26^ we hypothesised that MI activates neuroinflammatory mechanisms that down-regulate hippocampal neuron CFTR expression. We utilised lumacaftor, a therapeutic agent that increases CFTR expression via a direct proteostatic increase of the protein's half-life,^32^ as an intervention to counteract this down-regulation.^25^ Since lumacaftor is not clinically available as a monotherapy, we additionally tested a lumacaftor/ivacaftor combination therapy that is clinically approved and available. The additional ingredient in the combination therapy (ivacaftor) “potentiates” CFTR channel activity by extending the channel's open time following activation.^33^ We hypothesised that therapeutically correcting CFTR expression/activity would mitigate the MI-associated neurological defects in the hippocampus.

## Methods

### Materials

All chemical reagents and solutions were purchased from Fisher Scientific (Gothenburg, Sweden), Saveen & Werner (Limhamn, Sweden), Sigma–Aldrich (Stockholm, Sweden), or Nordic Biosite (Täby, Sweden) unless otherwise stated. Primers for qPCR were purchased from Eurofins (Ebersberg, Germany), Qiagen (Hilden, Germany), or PrimerDesign (Camberley, UK).

### Ethics approval

This investigation conforms to the EU Directive 2010/63/EU for animal experiments and the ARRIVE 2.0 guidelines. The study was performed according to the Swedish Animal welfare ACT SFS 1988:534 act, was approved by the institutional ethical committee for animal experiments Malmö/Lund (Dnr 5.8.18-08003/2017, 5.8.18-04938/2021) and conducted in accordance with European animal protection laws. Experimental work conducted at the University of Toronto was approved by the Institutional Animal Care and Use Committee at the University of Toronto (Approval # 20011425) and conducted in accordance with Canadian animal protection laws.

### Animals

This study utilised male wild-type (WT) C57Bl/6N mice (RRID:MGI:5756053) purchased from Taconic Biosciences (Ejby, Denmark) or Charles River Laboratories (Montreal, Quebec, Canada). Animals were housed in a climate-controlled facility under a standard 12 h:12 h light–dark cycle, fed normal chow and had access to food and water *ad libitum*. All mice were the same age and had comparable weights. Issues with immune status or health, genetic modification and genotype are not applicable to our study. Upon arrival, animals were acclimatised during 5 days before any handling or experimental procedure was initiated. Surgical procedures were conducted on 12-week-old mice with a body weight ≥25 g. Animals were randomly allocated to sham or MI groups using the computer software *Research Randomizer* (http://www.randomizer.org/).

### Induction of myocardial infarction in mice

MI was induced by surgical ligation of the left anterior descending (LAD) coronary artery as described previously.^34^ Briefly, mice were anaesthetised with 2–3% isoflurane (IsoFlo in room air; Abbott, Solna, Sweden), intubated with a 22-gauge angiocatheter (BD, Helsingborg, Sweden), and ventilated at a rate of 120 breaths per minute with a 200–250 μl tidal volume and 3 cm positive end expiratory pressure (MiniVent; Hugo Sachs, March, Germany). A left lateral thoracotomy was performed to expose the heart: the pericardium was opened, and the LAD was permanently ligated with 7-0 non-absorbable suture (AgnTho's; Lidingö, Sweden). Following ligation, the chest was closed, and mice were extubated after restoration of spontaneous respiration. Sham-operated control mice underwent an identical surgical procedure without LAD ligation. All mice received buprenorphine for post-surgical analgesia (0.05 mg/kg delivered subcutaneously every 12 h for 2–4 times).

Following surgery, mice were housed in groups of 3–5 animals per cage. At 9–10 weeks post–MI, cardiac ejection fraction (EF) was measured using magnetic resonance imaging (MRI) or echocardiography and MI mice were allocated to the following blocks: I) EF <30%, II) EF 31–40% and III) EF 41–50%. The MI animals were then assigned to either the treatment or the vehicle group, ensuring that each group (i) included animals from different EF blocks; and (ii) possessed comparable mean EF values. Animals were assigned into the following treatment groups at 10 weeks post-surgery: (i) MI + vehicle (ctrl; 10% DMSO in 50:50 PEG/H_2_O); (ii) MI + Lumacaftor (3 mg/kg in 10% DMSO in 50:50 PEG/H_2_O); or (iii) MI + Lumacaftor/Ivacaftor (3 mg/kg Lumacaftor/1.875 mg/kg Ivacaftor in 10% DMSO/50:50 PEG/H_2_O). The treatments were administered by intraperitoneal injection daily for 2 weeks, starting 10 or 22 weeks after MI induction. At endpoint, mice were euthanised under 2% isoflurane anaesthesia for tissue collection.

### Assessment of cardiac parameters

Cardiac function was assessed using MRI on a 9.4 T MR horizontal MR scanner equipped with Bruker BioSpec AVIII electronics, a quadrature volume resonator coil (112/087) for transmission and a 20 mm linear surface loop coil for reception (Bruker, Ettlingen, Germany), operating with ParaVision 6.0.1. as previously described.^34^ During imaging, mice were immobilised using 1.5–2.3% isoflurane in room air, supplemented with 10% oxygen and kept at a respiration of 70–100 bpm and at 36–37 °C body temperature. Flow compensated FLASH with electrocardiogram (ECG) and respiration triggering (Stony Brook, USA) with a resolution of 0.13 × 0.13 × 1 mm^3^ was used for all MR scans. Positioning of the cardiac images was achieved by three orientational scans: (1) three axial slices (TR = 50 ms, TE = 2.5 ms), (2) and (3) each with one slice (TR = 6 ms, TE = 2.1 ms, 24 timeframes) orthogonal to each other with slices positioned through the left and right ventricle and through the outflow tract of the left ventricle and the apex, respectively. Short axis view images of 9–10 slices (depending on heart size) were acquired with 24 timeframes in each (TR = 6 ms, TE = 2.1 ms). Hemodynamic parameters were assessed from the Dicom images using Segment (Medviso, Lund, Sweden).^35^ Left ventricular (LV) ejection fraction was calculated from LV-end-diastolic and LV-end-systolic dimensions.

Echocardiographic measurements were collected in isoflurane-anaesthetised mice with a GE Healthcare (Mississauga, Canada) Vivid 7 Dimension ultrasound system (i13L 14 Mhz linear-array transducer) or a VisualSonics (Toronto, Canada) Vevo 770 ultrasound system (30 MHz mechanical sector transducer), as previously described.^25^ Ventricular dimensions were measured in M-mode at the level of the papillary muscles.

Cardiac function parameters of all cohorts are presented in Supplemental Table S1.

### Novel object recognition test

Hippocampal non-spatial memory that involves the activation of cornu ammonis 1 (CA1) neurons^36^^,^^37^ was assessed using a novel object recognition task with a 1-h delay interval, as adapted from a previously described protocol.^38^ Mice were handled daily for seven days prior to acclimatisation to the experimental setup. Habituation to the behaviour boxes was realised through 8-min open field explorations on two consecutive days. Habituated mice were exposed to two identical objects for 8 min. On the test day, mice were re-exposed to the same two identical objects and after a 1-h delay, one of the original objects was replaced with a novel object and mice were allowed to explore the two objects for 8 min. The objects and arena were thoroughly cleaned with 70% ethanol between each test, to eliminate potential odour cues. Mice were video tracked with Any Maze software (Stoelting; Dublin, Ireland) that recorded the time spent interacting with the novel (Tn) and original (To) objects. The results were verified by manual Tn and To determination using stop watches by an observer blinded to the experimental group assignments. A recognition index (RI) was calculated from Tn/[Tn + To]. Animals with total exploration times below 20 s were excluded from analyses.

### Hippocampal perfusion measurements

MRI-based perfusion imaging was performed on a preclinical 9.4 T MRI scanner with Bruker BioSpec AVIII electronics operating with ParaVision 7.0.1. Mice were anaesthetised with 2.5% isoflurane (Vetflurane) with a mixture of room air: O2 (9:1); during the imaging the respiration was kept between 70 and 100 breaths/min, temperature was kept between 36 and 37 °C with 1.5–2.5% isoflurane. The head was fixed with a tooth bar. Mice were covered with a heating blanket to ensure constant body temperature (36–37 °C). Body temperature and respiration rate were controlled with the SA Instrument (Stony Brook) animal monitoring system. Arterial spin labelling (ASL) using Bruker FAIR-EPI with an echo time of 11 ms, spectral bandwidth of 217 kHz, partial Fourier acceleration factor of 1.5 in the phase direction, recovery time of 10 s and inversion times 0.03, 0.5, 1.0, 3.0, 5.0 and 9.7 s. A 90° pulse of 1.91 ms was used for excitation and a 180° pulse of 1.55 ms for refocusing, both with bandwidth of 2.2 kHz and a sharpness of 3,^39^ was used to assess CBF. One coronal slice was imaged per mouse. The resolution was 233 × 234 μm^2^, with field of view 17 × 15 μm^2^, slice thickness 1.5 mm. As orientation to identify hippocampal areas, T2-weighted images of the whole brain were acquired using Rapid Imaging with Refocused Echoes (RARE) sequence with repetition time = 3.5 s, echo time = 33 ms, 32 slices with 0.5 mm thickness, resolution of 100 × 100 μm^2^, field of view 16 × 16 mm^2^ and 2 averages. Regional CBF in the hippocampus was analysed in ImageJ and presented as ml/100 g ∗ min^−1^.

### Immunofluorescence analyses

Coronal brain sections (10 μm) or neuronal cells grown on poly-lysine pre-treated cover slides were stained with ionised calcium-binding adapter molecule-1 (Iba-1; FUJIFILM Wako Shibayagi Cat# 019-19741, RRID:AB_839504), CD68 (BioRad/Nordic Biosite Cat# MCA1957, RRID:AB_322219), apoptosis-associated speck-like protein containing a caspase-activation and recruitment domain/target of methylation-inducing gene silencing-1 (ASC/TMS1; inflammasome marker; Proteintech Cat# 67494-1-Ig, RRID:AB_2882718), Interleukin 33 (IL-33, R and D Systems Cat# AF3626, RRID:AB_884269), Interleukin 1β (IL-1β, Santa Cruz Biotechnology Cat# sc-52012, RRID:AB_629741), microtubule-associated protein 2 (MAP-2; neuronal marker; Abcam Cat# ab32454, RRID:AB_776174), or CFTR (Thermo Fisher Scientific Cat# MA1-935, RRID:AB_2081230; Supplemental Table S2) in a humidity chamber over night at 4 °C after blocking with blocking reagent (Roche). Slides were subsequently washed with phosphate buffered saline (PBS), incubated with secondary antibody (Supplemental Table S2) at room temperature, and mounted with Fluoromount-G with DAPI. In brain slices, dentate gyrus (DG) and CA1 hippocampus regions were assessed for CFTR+ neurons (positive for MAP-2) and Iba-1+ cells (microglia) positive for CD68 or ASC/TMS1 using a fluorescence microscope (Axio Imager M2, Zeiss).

All CA1 and DG Iba-1+ microglia were classified into ramified (resting state; cells with a small cell body and extensive branched processes), intermediate (cells with enlarged cell body and thickened, reduced branches not longer than twice the cell body length), and round (active state; cells with round cell body without visible branches).^38^ CFTR positive N2a cells and primary neurons were manually counted and expressed as % of total MAP-2+ cells.

### Histological analysis of dendrite morphology

Neuronal dendrite networks and dendritic spine density of pyramidal neurons of the hippocampus were assessed in coronal Golgi-Cox-stained brain sections (150 μm) as previously described.^26^^,^^40^ Neurons and their dendritic networks were imaged with a Nikon Eclipse Ti2 microscope (Nikon Instruments Europe) and analysed using Image J (https://imagej.net/Sholl_Analysis, version 3.7.4). The dendrite networks of single hippocampal neurons from CA1 region were digitally isolated from hyperstack images using the Simple Neurite Tracer plugin for Image J and analysed by Sholl analysis.^41^ The centre of the soma was pinpointed, and dendrite morphology was then characterised by Sholl analysis (https://imagej.net/Sholl_Analysis, version 3.7.4), using 5 μm intervals. Dendrite intersections (i.e., branching) and maximum dendrite length can be characterised by this Sholl analysis. For each treatment group, at least 2 pyramidal CA1 neurons per brain from 3 to 5 mice per group were analysed, under blinded conditions.

Dendritic spine density (i.e., the number of small protrusions found on dendrites) was assessed using 3rd branch order dendritic segments at 100× magnification. Spine density was measured as the number of spines per segment normalised to the length of the segment. For each treatment group, spine density was measured in 3 pyramidal CA1 neurons (2 third order branch per neuron) per mouse from 3 to 5 mice, under blinded conditions.

### Cell culture

Murine microglial cells (BV2, ATCC CRL-2469; RRID:CVCL_0182) and murine neuronal cells (Neuro-2a (N2a), ATCC CCL-131; RRID:CVCL_0470) were grown in Dulbecco's modified Eagle's medium (DMEM, Gibco) supplemented with 10% (v/v) foetal bovine serum (Gibco) and 1% (v/v) Penicillin-Streptomycin (10.000 U/ml; Gibco) until 80% confluency. Regular mycoplasma testing was performed. BV2 microglial cells were treated with vehicle or lipopolysaccharide (LPS; 100 ng/ml) for 48 h: the resulting supernatants were designated as conditioned medium (CM) or LPS-conditioned medium (LCM), respectively. Supernatants were frozen at −20 °C prior to co-culture experiments with N2a cells. N2a cells were incubated with culture media, CM, or LCM for 24 h. A subset of experiments used a 48-h treatment period: the media was renewed after 24 h, with the addition of 10 μM Lumacaftor or vehicle.

Primary embryonic neurons were prepared as previously described.^42^ Briefly, neurons were prepared from cortices and hippocampi of embryonic day 15–17 WT mouse embryos and cultured *in vitro* in Neurobasal medium supplemented with glutamine, B27 and penicillin-streptomycin (Gibco) for 19–21 days prior to staining.

### Fluorescence activated cell sorting

After culture media removal, cells were collected by washing the wells with PBS. The cell-PBS solution was centrifuged at 400×g and 4 °C for 5 min, the supernatant was removed, and the cell pellet was resuspended in fluorescence-activated cell sorting (FACS) buffer (PBS + 2% FBS + 2 mM ethylenediaminetetraacetic acid (EDTA); pH 7.4). Samples were stained with CFTR antibody labelled with Alexa Fluor-647 NHS ester for 30 min at 4 °C. Samples were prepared for acquisition by removing antibody solution after washing with FACS buffer and centrifugation at 400×g at 4 °C for 5 min prior to resuspension in FACS buffer. Data acquisition was carried out in a BD Accuri C6 Plus cytometer (BD Biosciences). Data analysis was performed with FlowJo software (version 10, TreeStar Inc., USA). Cells were plotted on forward versus side scatter and single cells were gated on FSC-A versus FSC-H linearity. Median statistic was added to the gated population of CFTR+ cells.^34^

### Western blotting

Hippocampal tissue samples were homogenised in 1× PBS using an Ultra-Turrax TP18-10 (Janke & Kunkel KG) and then solubilised in lysis buffer (10 mM Tris pH 8.0, 1 mM EDTA, 1% Triton-X, 0.1% sodium-deoxycholate, 0.1% SDS, 140 mM NaCl) supplemented with phosphatase and protease inhibitors for 30 min on ice. To remove insoluble material, the protein extracts were centrifuged for 10 min at 20,000×g at 4 °C. The supernatants were then stored at −20 °C until further processing. An identical lysis solubilisation procedure was executed for N2a cells. Protein content was measured using the Pierce BCA Protein Assay Kit according to manufacturer's instructions. Proteins were electrophoretically separated on density gradient polyacrylamide gels and transferred onto polyvinylidene difluoride membranes (GE Healthcare; Uppsala, Sweden) using semi-dry transfer in TransBlot Turbo (Bio-Rad).

Following transfer, the membranes were blocked in PBS containing 0.05% Tween 20 (PBS-T) and 5% non-fat dry milk for 1 h at room temperature. Membranes were subsequently incubated with primary antibody (Supplemental Table S3) in 5% non-fat dry milk in PBS-T overnight at 4 °C. After primary antibody incubation, the membranes were washed 5× in PBS-T and incubated in secondary horseradish peroxidase (HRP)-conjugated anti-mouse or anti-rabbit antibody (Supplemental Table S3) for 1 h at room temperature in PBS-T. The membranes were then washed 5× with PBS-T and visualised by enhanced chemiluminescence using a ChemiDoc MP (Bio-Rad). As a loading control, membranes were probed with primary mouse anti-tubulin or mouse anti-actin for 1 h at room temperature and sequentially incubated with HRP-conjugated anti-mouse antibody for 1 h at room temperature in PBS-T. Protein expression was then detected as described above.

### Quantitative real-time PCR

Total RNA was extracted from N2a and BV2 cells and hippocampal brain homogenates using TriZol Reagent (Invitrogen) according to manufacturer's instructions. RNA concentration and quality were determined with a NanoDrop 2000c spectrophotometer (Thermo Fisher Scientific). 1 μg of total RNA was reverse transcribed with random hexamer primers using a “High-Capacity Reverse Transcriptase Kit” kit (Applied Biosystems; Gothenburg, Sweden). The resulting cDNA was diluted with RNAse-free water (1:12.5) and used as template for quantitative real-time PCR (qRT-PCR) reactions. The following primers were used:

Ribosomal Protein L14 (L14): (fwd: GGCTTTAGTGGATGGACCCT, rev: ATTGATATCCGCCTTCTCCC); inducible nitric oxide synthase (iNOS) (fwd: GGCAGCCTGTGAGACCTTTG, rev: GCATTGGAAGTGAAGCGTTTC); tumour necrosis factor (TNF) (fwd: CCCTCACACTCACAAACCACC, rev: GCCTTGTCCCTTGAAGAGAAC); IL-1β (fwd: GAAGAGCCCATCCTCTGTGA, rev: TTCATCTCGGAGCCTGTAGTG); Arginase-1 (Arg-1) (fwd: TTGCGAGACGTAGACCCTGG, rev: CAAAGCTCAGGTGAATCGGC); post-synaptic density protein 95 (PSD-95) (fwd: ACCGCTACCAAGATGAAGACAC, rev: CTCCTCATACTCCATCTCCCC); Interleukin 18 (IL-18) (fwd: TGAGGCATCCAGGACAAATCAG, rev: AGCACACCACAGGGGAGAAG).

Gene expression was measured with qRT-PCR using Fast SYBR Green (Applied Biosystems, Naerum, Denmark) and 0.2 μM gene specific forward and reverse primers in a CFX96 TouchTM Real-Time PCR Detection System (Bio-Rad; Sundbyberg, Sweden). The PCR cycle parameters were: 95 °C for 10 min, a total of 39 cycles (95 °C for 15 s, 60 °C for 1 min) followed by a dissociation stage (95 °C for 1 min, 55 °C for 70 s and 95 °C for 50 s). Data were analysed using the absolute method using standard curves generated from pooled cDNA representative of each sample to be analysed and normalised to the housekeeping gene Ribosomal Protein L14.

Gene expression of functional Interleukin-8 homologue (Qiagen, Mm_Cxcl1_SG QuantiTect Primer Assay (QT00115647)), IL-33 (Primerdesign, NM_001164724, Sense primer: CAATGTTGACGACTCTGGAAAAG, antisense: GGGACTCATGTTCACCATCAG), and β-actin (Qiagen, Mm_Actb_1_SG QuantiTect Primer Assay (QT00095242)) was measured on a Mx3005P qPCR system (AriaMx, Agilent) using QuantiNova master mix (Qiagen) with the following cycle parameters: 95 °C for 2 min followed by a total of 40 cycles (95 °C for 5 s, 60 °C for 22 s). Samples were analysed by the ΔCt method and normalised to β-actin expression.

### Statistics

Using previous data as guidance,^25^^,^^26^^,^^43^^,^^44^ we calculated the experimental group sizes necessary to ensure that all data provide an 80% power level (1 − β > 0.8) and a two-tailed Type I alpha error of 0.05. Since behavioural testing requires the highest sample size, we completed an a priori sample size calculation (performed with G∗Power; https://www.psychologie.hhu.de/arbeitsgruppen/allgemeine-psychologie-und-arbeitspsychologie/gpower) using previously published novel object recognition index data (sham = 0.68 ± 0.10; MI = 0.49 ± 0.14).^2^^,^^10^ The calculation determined that a minimum of 8 animals per group (d = 1.56; Df = 13.28) are required for adequate statistical power. Due to model-specific mortality, 20% attrition was added per group.

This study is comprised of 5 separate MI cohorts: [1] 3 months post–MI without treatment (N = 9 sham and N = 9 MI); [2] 3 months post–MI with Lumacaftor treatment (N = 10 sham, N = 10 MI + vehicle, N = 10 MI + Lum); [3] 3 months post–MI with Lumacaftor/Ivacaftor treatment (N = 10 sham, N = 9 MI + vehicle, N = 10 MI + Lum/Iva); [4] 6 months post–MI with Lumacaftor/Ivacaftor treatment (N = 8 sham, N = 8 MI + vehicle, N = 8 MI + Lum/Iva), and [5] a longitudinal MI cohort without treatment (N = 10 MI). An overview of the experimental setup is shown in Supplemental Fig. S1.

All assessments and analyses in this study were conducted under blinded conditions, using identification codes that concealed the identity of the intervention. Data were analysed using GraphPad Prism 9 software (San Diego, California). Normally distributed data are presented as mean ± SEM and compared using parametric statistical tests. Data that are not normally distributed, as determined by the Shapiro–Wilk test, are presented as median ± interquartile range and are compared using non-parametric statistical tests. A Student's t-test or Mann Whitney test was used to compare two independent groups; a one-way analysis of variance (ANOVA) with Tukey *post-hoc* test or Kruskal Wallis test with Dunn's *post-hoc* test was used to compare multiple independent groups. Differences are considered significant at p ≤ 0.05. For all data sets, N represents the number of animals or independent biological sample and n the number of independent measures. All figure data, sample size, and statistical test outcomes are presented in Supplemental Tables S4 and S5.

### Role of the funding source

The study sponsors played no role in the study design; the collection, analysis, and interpretation of the data; manuscript preparation; or the decision to submit for publication.

## Results

**Fig. 1:**
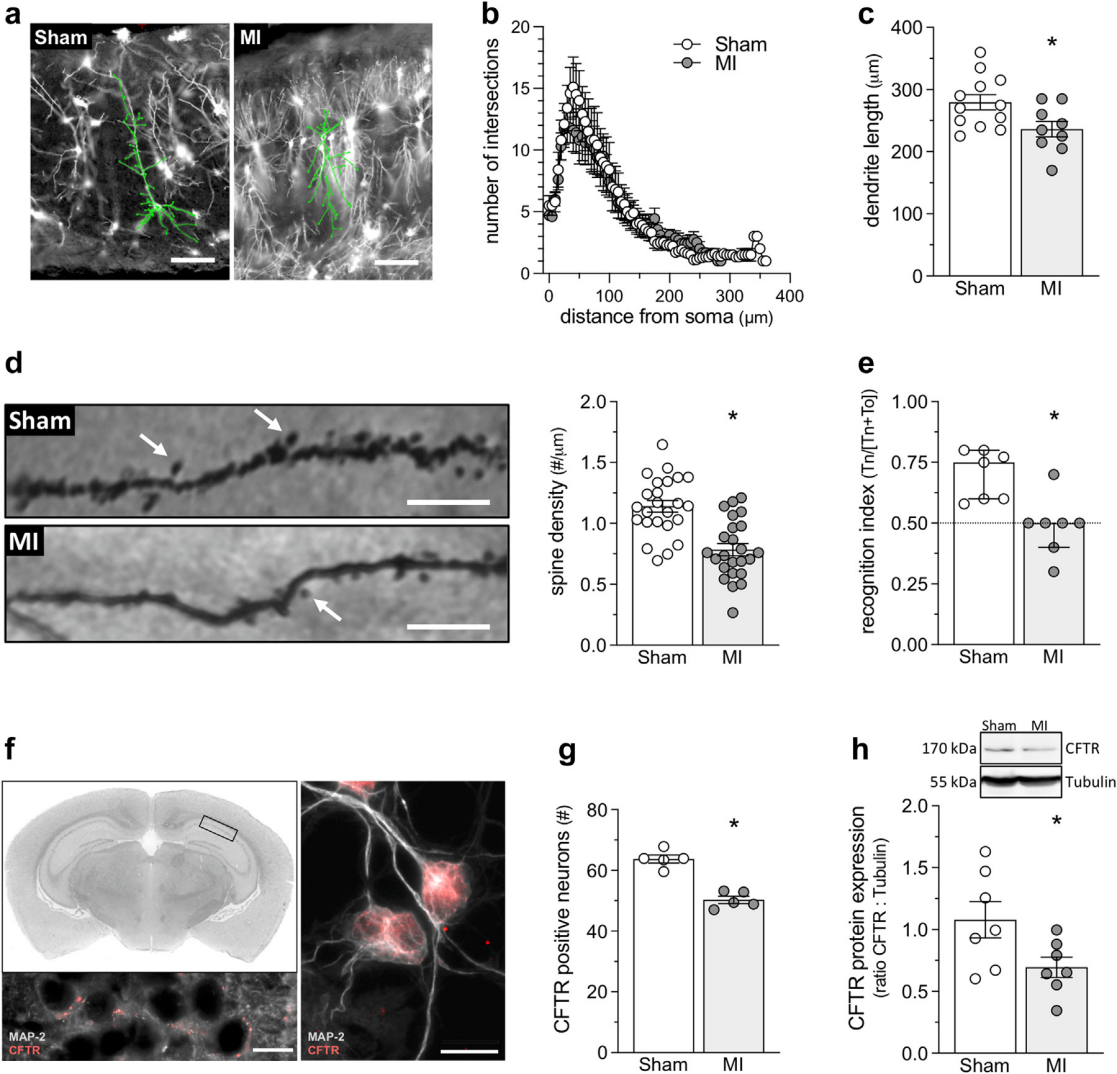
**Neuronal morphology and function following myocardial infarction.(a)** Shown are representative images of Golgi-stained hippocampal cornu ammonis (CA1) neurons from mice at 12 weeks post-sham or post-myocardial infarction (MI). The dendrite's morphology is highlighted with traces superimposed onto the images. **(b)** Sholl analysis histograms plotting the number of dendrite intersections (i.e., dendritic branching) versus dendrite length (i.e., distance from neuronal soma) show no qualitative differences in branching morphology between the 2 groups. However, **(c)** mean dendrite length is significantly reduced in MI mice relative to sham mice (n = 9 neurons from N = 3 mice for MI and n = 12 neurons from N = 4 for Sham). **(d)** Shown are representative images of dendritic spines in hippocampal CA1 neurons and the quantification of the number of spines per micrometre on tertiary branches of the pyramidal cells. MI reduces dendrite spine density (n = 24 analysed branches from N = 4 mice). **(e)** MI attenuates hippocampal-dependent retention of object familiarity in a non-spatial novel object recognition task (N = 7; 3 mice from sham and 1 mouse from MI groups were excluded due to total exploration times below 20 s). **(f)** Hippocampal neurons express the cystic fibrosis transmembrane conductance regulator (CFTR). The top-left image shows a coronal brain slice with a hippocampal region marked for cell isolation or immunostaining analysis. The bottom left image displays representative co-immunostaining for CFTR and microtubule-associated protein 2 (MAP-2; neuronal marker) in CA1 hippocampal neurons within brain slices. The right-hand image displays CFTR and MAP-2 co-immunostaining in cultured primary neurons. **(g)** MI reduces the number of hippocampal CA1 and dentate gyrus (DG) neurons positively stained for CFTR (n = 5 sections from N = 3 mice). **(h)** MI reduces CFTR protein expression in hippocampal lysates (Western blot; N = 7). ∗ denotes p < 0.05 for unpaired comparisons. Panels c, d, g, and h are presented as mean ± SEM and are compared with Student's t-test; Panel e is presented as median ± interquartile range and is compared with a Mann–Whitney test. Bar over micrographs is 120 μm in Panel a, 5 μm in Panel d and 10 μm in Panel f.

Mouse hippocampal neuron dendrite length (Fig. 1a–c, p = 0.025; t-test) and dendritic spine density (Fig. 1d, p < 0.001; t-test) are reduced at 12 weeks post–MI, consistent with previous observations in cortical regions.^25^ In contrast, no obvious differences in dendrite branching (i.e., the number on intersections at a given distance from the soma) are evident (Fig. 1b). At the functional level, novel object recognition tests demonstrate non-spatial long-term memory impairment compared to sham-operated controls (Fig. 1e, p = 0.002; Mann–Whitney). Concomitantly, hippocampal CFTR expression is reduced, as measured by the number of CFTR-expressing neurons identified in the CA1 and DG (Fig. 1f–g, p < 0.001; t-test), hippocampal regions responsible for non-spatial memory and integration of entorhinal cortex signals,^36^^,^^37^^,^^45^ and western blots of hippocampal lysates (Fig. 1h, p = 0.042; t-test). In contrast to our previously published cortical perfusion measurements,^25^ longitudinal assessment of hippocampal blood flow revealed no significant changes at 12-weeks post–MI compared to baseline (Supplemental Fig. S2, p = 0.4316; Wilcoxon signed rank).

**Fig. 2:**
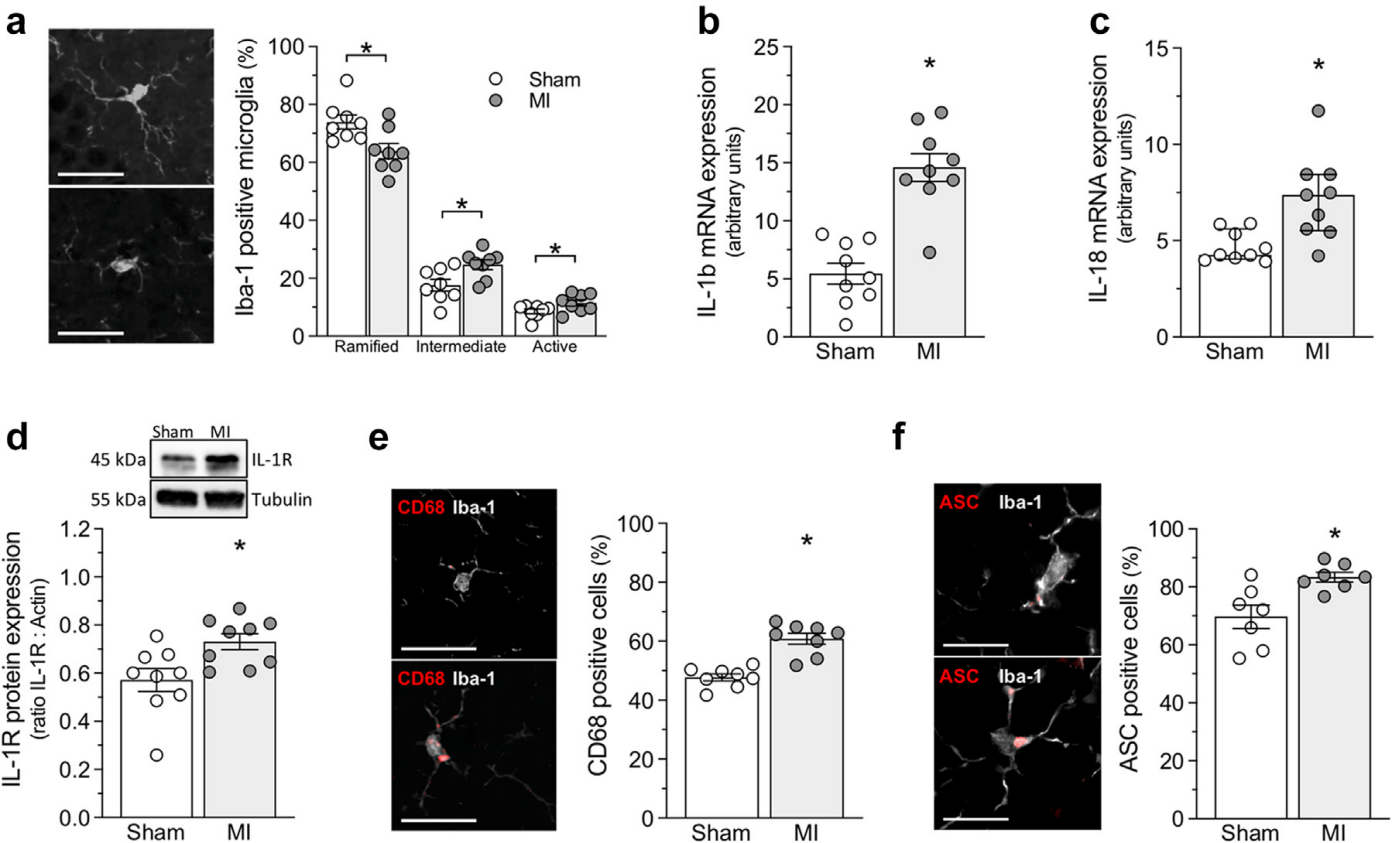
**Microglial activation in the hippocampus following myocardial infarction.(a)** Shown are representative immunofluorescence images of hippocampal cornu ammonis (CA1) microglia stained for ionised calcium-binding adapter molecule-1 (Iba-1); Iba-1 positive microglia were classified as ramified (top), intermediate (bottom), or active (not shown) based on their morphology in CA1 and dentate gyrus (DG) hippocampus regions. Myocardial infarction (MI) decreased the proportion of ramified microglia and increased the proportion of intermediate and active microglia (n = 8 sections from N = 4 mice). MI increases **(b)** interleukin-1β (IL-1β; N = 9) and **(c)** interleukin-18 (IL-18; N = 9) mRNA expression in the hippocampus; **(d)** hippocampal IL-1 receptor (IL-1R) protein expression is also increased following MI (N = 9). Panels e and f display representative immunofluorescence images of CA1 hippocampal microglia co-stained for Iba-1 (white) and **(e)** CD68 (red) or (f) apoptosis-associated speck-like protein containing a caspase-activation and recruitment domain (ASC; red); low (top) and high (bottom) abundance CD68 and ASC signals are shown. MI increases the proportion of (e) CD68-positive microglia (n = 8 sections from N = 4) and **(f)** ASC-positive microglia (n = 7 sections from N = 4) in CA1 and DG hippocampus regions. ∗ denotes p < 0.05 for unpaired comparisons. Panels a, b, d, e, and f are presented as mean ± SEM and are compared with Student's t-test; Panel c is presented as median ± interquartile range and is compared with a Mann–Whitney test. Bar over micrographs is 50 μm in Panels a and e and 10 μm in Panel f.

Microglia, identified by positive Iba-1 staining, are more activated in MI mice, as evidenced by the reduced proportion of ramified morphology (associated with quiescent cells, p = 0.014; t-test) in favour of the “intermediate” (associated with activation, p = 0.016; t-test) and “active” (associated with scavenging, p = 0.048; t-test) morphologies in CA1 and DG regions of the hippocampus (Fig. 2a). Consistent with this morphological indicator, molecular markers of inflammation and microglial activation are elevated in MI mice. Specifically, hippocampal lysates from MI mice display higher expression of IL-1β (p < 0.001; t-test), IL-18 (p = 0.006; Mann–Whitney), IL-8 homologue (KC(CXCL1, p = 0.001; Mann–Whitney)), and IL-33 (IL-33, p = 0.029; t-test) mRNA and higher IL-1β receptor (IL-1R, p = 0.014; t-test) protein expression than sham controls (Fig. 2b–d and Supplemental Fig. S3a and b). Likewise, IL-33 protein abundance is greater in the MI hippocampus compared to sham (Supplemental Fig. S3c; p = 0.006; t-test). Specifically, microglia IL-33 positivity is higher in the hippocampus of MI mice compared to sham operated controls (Supplemental Fig. S3d, p = 0.002; t-test). Immunofluorescence further confirmed IL-1β abundance in MI microglia (Supplemental Fig. S3e).

Following MI, microglial cells residing in CA1 and DG regions of the hippocampus display augmented expression of CD68, which is considered a marker of activated phagocytic microglia^46^ (Fig. 2e, p < 0.001; t-test). Moreover, CA1 and DG hippocampal microglia of MI mice present with higher levels of inflammasome complex related proteins, including apoptosis-associated speck-like protein containing a caspase-activation and recruitment domain (ASC; Fig. 2f, p = 0.008; t-test).

### *In vitro* studies linking microglial activation to neuronal CFTR down-regulation and injury

**Fig. 3:**
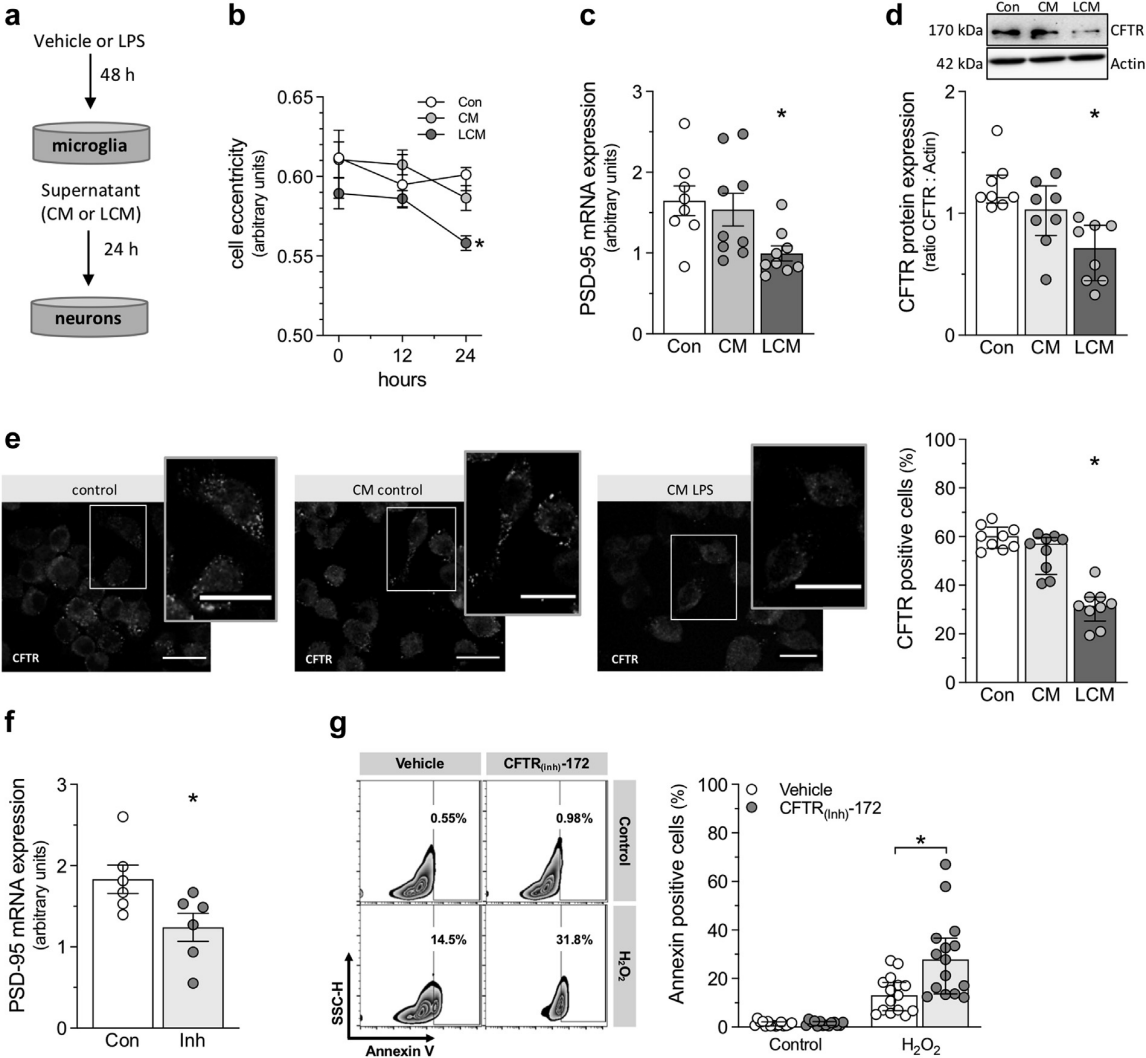
**Microglial activation reduces N2a neuronal cell CFTR expression *in vitro*.(a)** Shown is a pictogram illustrating the experimental design. BV2 microglial cells were treated with vehicle or lipopolysaccharide (LPS, 100 ng/ml)) for 48 h; the resulting supernatants were designated as conditioned medium (CM) or LPS-conditioned medium (LCM), respectively. The media were then applied to N2a neuronal cell cultures for 24 h and compared to controls (Con) that did not receive conditioned medium. LCM, but not CM, reduced N2a neuronal cell **(b)** eccentricity (n = 15 from 5 independent experiments), **(c)** post-synaptic density protein 95 (PSD-95) mRNA expression (n = 8–9 from 4 independent experiments; 1 sample in the control group did not yield sufficient RNA) and **(d)** cystic fibrosis transmembrane conductance regulator (CFTR) protein expression (n = 8 from 4 independent experiments). **(e)** Shown are representative immunofluorescence images of N2a neuronal cells stained for CFTR following treatment with either control medium, CM or LCM (Bar over micrographs is 10 μm). LCM, but not CM, reduced the proportion of CFTR positive cells (n = 9). **(f)** Inhibiting N2a neuronal cell CFTR channel activity (10 μM CFTR_(inh)_-172, 48 h; Inh) down-regulates PSD-95 mRNA expression (n = 6 from 3 independent experiments). **(g)** Shown are representative fluorescence activated cell sorting profiles of side scatter height (SSC-H) and Annexin V immunofluorescence. The upper limit of the Annexin V signal in unstained control cells was set as the threshold (vertical line). Cells crossing this threshold were quantified relative to the total population. Inhibiting CFTR channel activity (10 μM CFTR_(inh)_-172, 48 h) increases N2a neuronal cell injury to a cytotoxic H_2_O_2_ challenge (*n* = 14–15 from 5 independent experiments; 1 sample in the CFTR_(inh)_-172 group could not be analysed). ∗ denotes p < 0.05 for unpaired comparisons; Panels c and f are presented as mean ± SEM. Panels d, e, g, and the control curve in Panel b are presented as median ± interquartile range. In Panel b, comparisons to respective 0-h time point tested with Kruskal–Wallis (Con) or ANOVA + Dunnett's (CM and LCM); in Panel c, comparisons are tested with ANOVA + Dunnett's; in Panel d and e with Kruskal–Wallis + Dunn's; in Panel f with Student's t-test; and in Panel g, comparisons are tested to respective vehicle-treated cells with Mann–Whitney.

LPS (100 ng/ml LPS; 48 h) activates BV2 microglia *in vitro*, as evidenced by the increased mRNA expression of the classical activation markers: iNOS (p < 0.001; Mann–Whitney), TNF (p = 0.004; Mann–Whitney), and IL-1β (p < 0.001; Welch's test) (Supplemental Fig. S4a–c).^47^ The up-regulation of iNOS mRNA, coupled with the down-regulation of the M2 phenotype marker Arg-1 (p < 0.001; Mann–Whitney) (Supplemental Fig. S4d), is indicative that LPS induces a proinflammatory M1 phenotype.^48^^,^^49^ Conditioned medium collected from non-stimulated microglia (CM) (Fig. 3a) does not affect N2a neuronal cell morphology (cell eccentricity; Fig. 3b, p = 0.117; ANOVA) or the mRNA expression of PSD-95, a key regulatory protein that controls the maturation, size, and strength of synapses^50^^,^^51^ (Fig. 3c, p = 0.853; ANOVA + Dunnett's). In contrast, conditioned medium collected from LPS-stimulated microglia (LCM) reduces N2a neuronal cell eccentricity (Fig. 3b, p = 0.005; ANOVA + Dunnett's) and the expression of PSD-95 (Fig. 3c, p = 0.021; ANOVA + Dunnett's). These alterations are consistent with inflammatory damage/stress. Conditioned medium collected from LPS-stimulated microglia profoundly reduces N2a neuronal cell CFTR protein expression, as measured by Western blot (Fig. 3d, p = 0.001; Kruskal–Wallis + Dunn's) and the number of CFTR-positive cells (Fig. 3e, p < 0.001; Kruskal–Wallis + Dunn's). Neither conditioned medium from unstimulated microglia nor directly treating N2a neuronal cells with LPS down-regulates N2a neuronal cell CFTR expression (Fig. 3d and e, p = 0.223; Kruskal–Wallis + Dunn's – p = 0.597; Kruskal–Wallis + Dunn's, and Supplemental Fig. S5, p = 0.510; Mann–Whitney). Thus, cytokines released into the conditioned medium following microglial activation (Supplemental Fig. S6) are likely responsible for down-regulating CFTR expression in N2a neuronal cells. Intriguingly, acutely inhibiting CFTR channel activity (10 μM CFTR (inh)-172, 24 h) mediates a significant down-regulation of PSD-95 in the absence of LPS-stimulated conditioned medium (Fig. 3f, p = 0.037; t-test). Collectively, these results suggest that the loss of CFTR activity is detrimental to N2a cell health. Accordingly, while CFTR inhibition does not overtly augment N2a neuronal cell apoptosis (assessed by Annexin V staining), it increases the cytotoxicity of a H_2_O_2_ challenge (Fig. 3g, p = 0.003; Mann–Whitney).

**Fig. 4:**
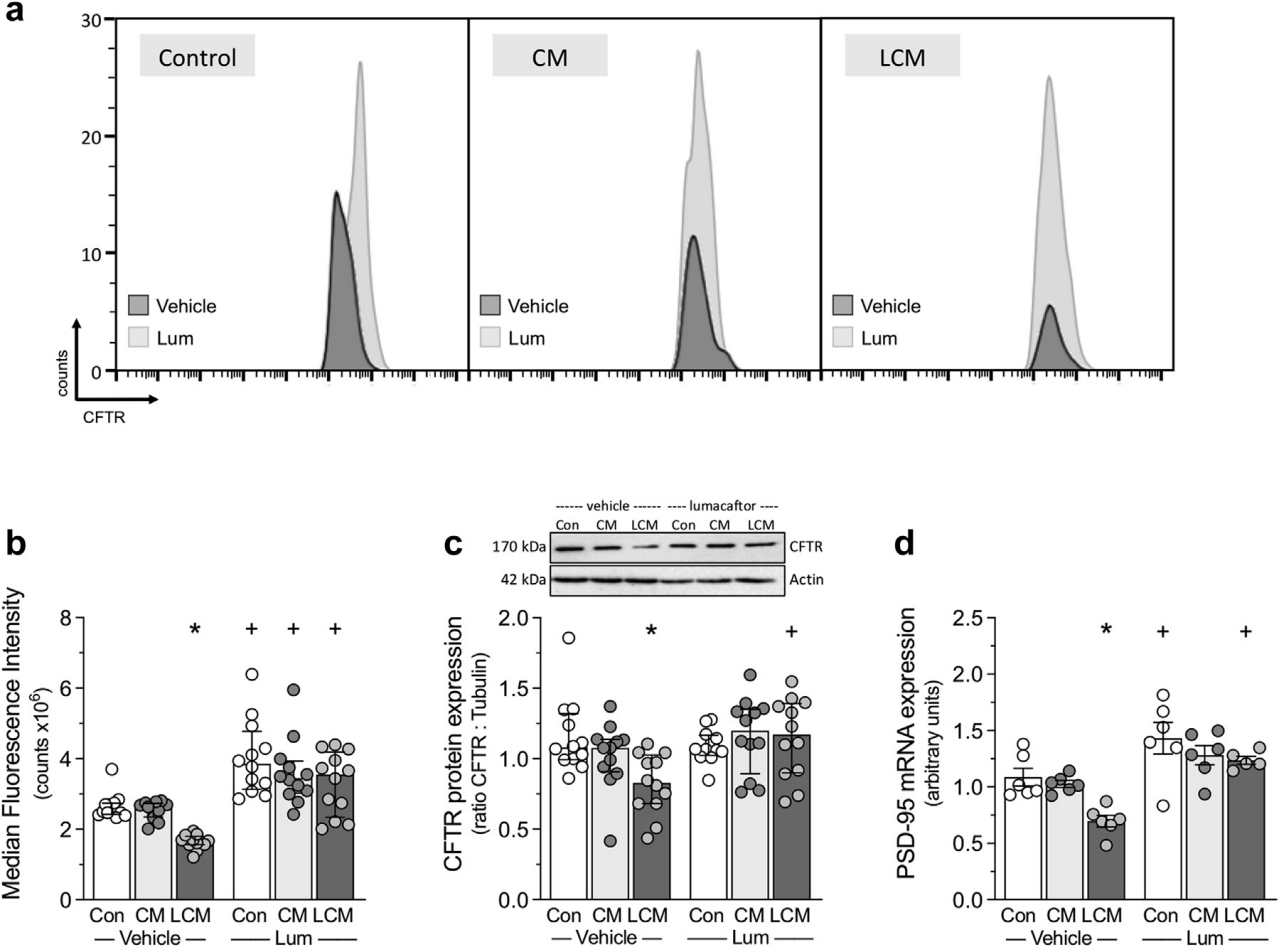
**Lumacaftor rescues neuronal CFTR expression following microglial activation *in vitro*.** BV2 microglial cells were treated with vehicle or lipopolysaccharide (LPS, 100 ng/ml)) for 48 h; the resulting supernatants were designated as conditioned medium (CM) or LPS-conditioned medium (LCM), respectively. Media were then applied to N2a neuronal cell cultures for 48 h, with 10 μM lumacaftor applied during the final 24 h period. **(a)** Representative flow cytometry histograms displaying median fluorescence intensity (MFI) for cystic fibrosis transmembrane conductance regulator (CFTR) cell surface expression in vehicle and lumacaftor-treated N2a neuronal cells. LCM, but not CM, reduces **(b)** CFTR MFI (n = 12 from 4 independent experiments), **(c)** CFTR protein expression (assessed via Western blot; n = 12 from 4 independent experiments) and **(d)** PSD-95 mRNA expression (n = 6 from 3 independent experiments). Lumacaftor treatment reverses these LCM-mediated reductions (n = 5 from 3 independent experiments; 1 sampled from the Lum group did not yield sufficient RNA). ∗ denotes p < 0.05 for unpaired comparisons to the respective vehicle- or lumacaftor-treated control; + denotes p < 0.05 comparing the effect of lumacaftor versus vehicle for Con, CM, and LCM treatment groups. Panels b and c are presented as median ± interquartile range and are compared with non-parametric statistical tests (Kruskal–Wallis + Dunn's). Panel d is presented as mean ± SEM and is compared with parametric statistical testing (ANOVA + Bonferroni).

Lumacaftor treatment (10 μM, 24 h) increases CFTR expression on the cell surface of CFTR^+^ N2a neuronal cells, as evidenced by increased median fluorescence intensity (Fig. 4a and b, p = 0.010; Kruskal–Wallis + Dunn's) and normalises the LCM-mediated reduction in CFTR protein, as assessed via Western blot (Fig. 4c, p = 0.010; Kruskal–Wallis + Dunn's). Likewise, Lumacaftor treatment rescues the LCM-mediated PSD-95 down-regulation (Fig. 4d, p = 0.001; ANOVA + Bonferroni). Intriguingly, Lumacaftor treatment increases PSD-95 expression in control cells (Fig. 4d, p = 0.034; ANOVA + Bonferroni).

### Therapeutic CFTR correction reverses neuronal injury following myocardial infarction

**Fig. 5:**
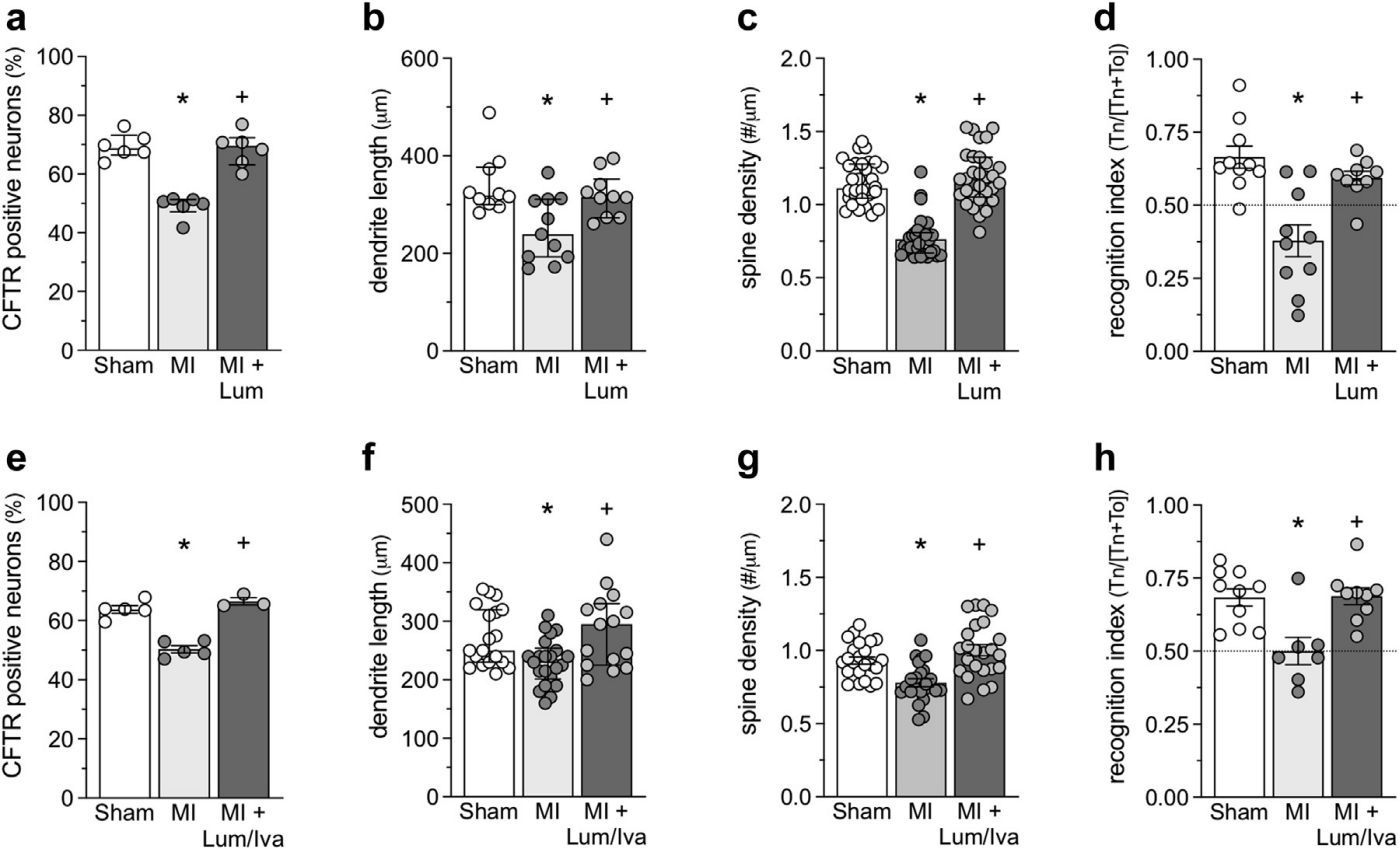
**CFTR therapeutics normalise neuronal morphology and function following myocardial infarction.** Lumacaftor treatment (Lum; 3 mg/kg daily for 2 weeks, starting at 10 weeks post-myocardial infarction [MI]) normalises the MI-induced reduction in **(a)** CA1 and DG neurons positively stained for CFTR (n = 6 sections from N = 3 mice), **(b)** pyramidal CA1 neuron mean dendrite length (n = 10–11 neurons from N = 4 mice) and **(c)** pyramidal CA1 neuron dendrite spine density (n = 30 analysed branches from N = 5 mice) in the hippocampus. **(d)** Functionally, lumacaftor treatment normalises hippocampal-dependent retention of object familiarity in a non-spatial novel object recognition task (N = 9–10; 1 mouse from the MI + Lum group was excluded due to total exploration times below 20 s). Likewise, lumacaftor/ivacaftor combination therapy (Lum/Iva; 3 mg/kg/1.875 mg/kg daily for 2 weeks, starting at 10 weeks post–MI) also normalises the MI-induced reduction in **(e)** CA1 and DG neurons positively stained for CFTR (n = 3–5 sections from N = 3 mice), **(f)** pyramidal CA1 neuron mean dendrite length (n = 15–22 neurons from N = 4 mice), **(g)** pyramidal CA1 neuron dendrite spine density (n = 24 analysed branches from N = 4 mice) in the hippocampus; **(h)** Lum/Iva also normalises hippocampal-dependent retention of object familiarity in a non-spatial novel object recognition task (N = 7–10; 2 mice from MI + vehicle and 1 mouse from MI + Lum/Iva groups were excluded due to total exploration times below 20 s). ∗ denotes p < 0.05 for unpaired comparisons to the sham; + denotes p < 0.05 for an unpaired comparison between MI and Lum or MI and Lum/Iva. Panels a, b, c and f, are presented as median ± interquartile range and are compared with non-parametric statistical tests (Kruskal–Wallis + Dunn's). Panels de, g and h are presented as mean ± SEM and are compared with parametric statistical tests (ANOVA + Tukey's).

*In vivo*, lumacaftor treatment in mice following MI (3 mg/kg daily for 2 weeks, starting at 10 weeks post–MI) normalises hippocampal neuron CFTR expression (Fig. 5a, p = 0.011; Kruskal–Wallis + Dunn's), which associates with profound improvements in functional and histological parameters (Fig. 5b–d). Specifically, lumacaftor treatment normalises novel object recognition (Fig. 5d, p = 0.003; ANOVA + Tukey's) and at the histological level, eliminates the MI-induced reduction in hippocampal neuron dendrite length (Fig. 5b, p = 0.040; Kruskal–Wallis + Dunn's) and dendrite spine density (Fig. 5c, p < 0.001; Kruskal–Wallis + Dunn's).

Since lumacaftor is not clinically available as a monotherapy, we investigated whether an FDA-approved, clinically available lumacaftor/ivacaftor combination therapy (marketed as Orkambi®) confers the same therapeutic benefit as lumacaftor alone. Indeed, lumacaftor/ivacaftor combination therapy (3 mg/kg/1.875 mg/kg daily for 2 weeks, starting at 10 weeks post-myocardial infarction) normalises (i) hippocampal neuron CFTR expression (Fig. 5e, p < 0.001; ANOVA + Tukey's), (ii) hippocampal neuron dendrite length (Fig. 5f, p = 0.029; Kruskal–Wallis + Dunn's) and spine density (Fig. 5g, p < 0.001; ANOVA + Tukey's), and (iii) long-term memory, as measured by novel object recognition (Fig. 5h, p = 0.003; ANOVA + Tukey's) in mice at 12 weeks post–MI.

**Fig. 6:**
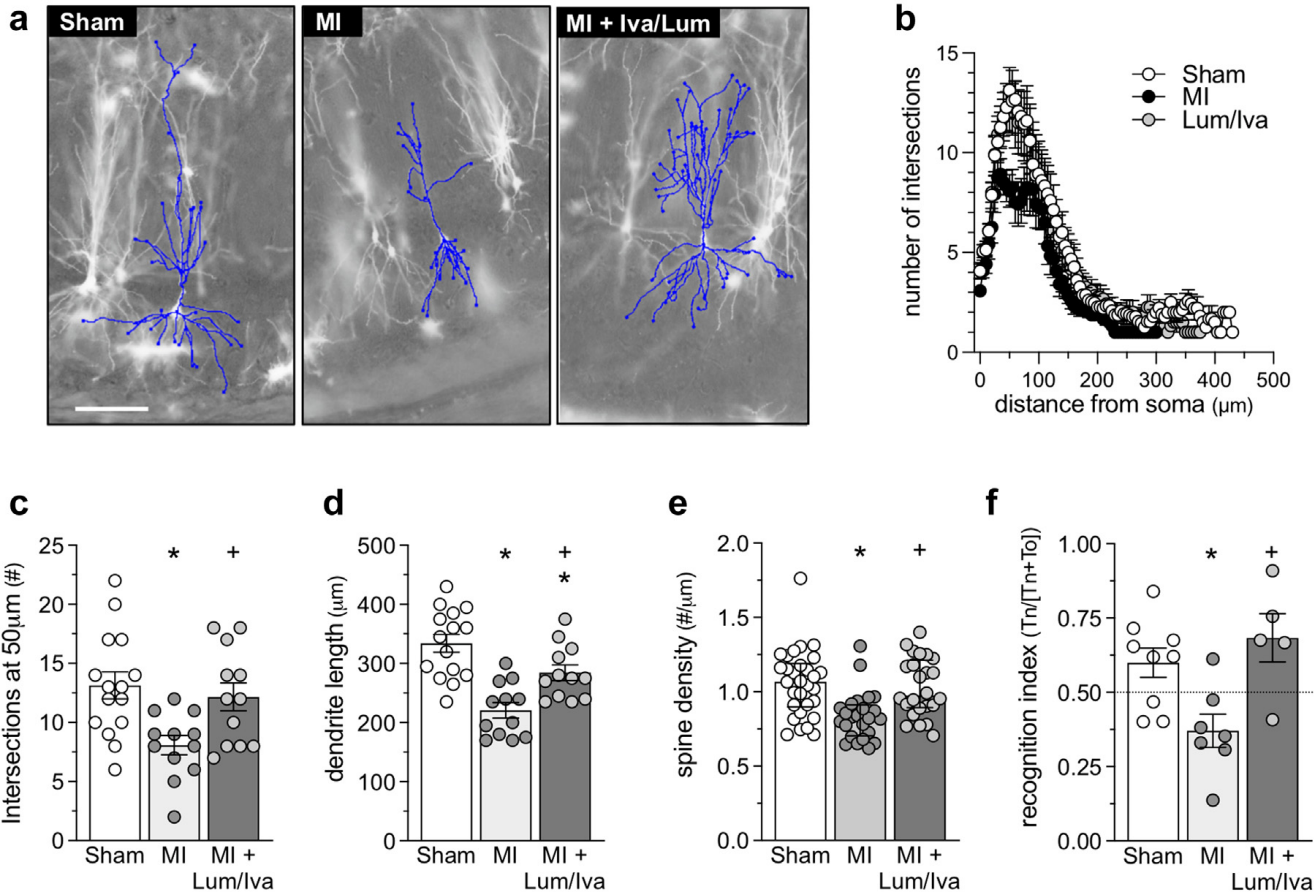
**CFTR therapeutics normalise neuronal morphology and function following extended treatment delay.(a)** Shown are representative images of Golgi-stained hippocampal CA1 neurons from mice at 6 months post-sham, post-myocardial infarction (MI) and post–MI with lumacaftor/ivacaftor combination therapy (Lum/Iva; 3 mg/kg/1.875 mg/kg daily for 2 weeks, starting at 22 weeks post–MI). The dendrite's morphology is highlighted with traces superimposed onto the images. **(b)** Sholl analysis histograms plotting the number of dendrite intersections (i.e., dendritic branching) versus dendrite length (i.e., distance from neuronal soma). Qualitatively, MI reduces the number of intersections between 40 and 100 μm and this defect is reversed by lumacaftor treatment. **(c)** MI reduces the number of intersections measured at 50 μm from the soma; Lum/Iva treatment attenuates this reduction (n = 15 neurons from N = 5 for sham and n = 12 neurons from N = 4 MI mice). **(d)** Mean dendrite length is reduced in MI mice relative to sham mice; Lum/Iva treatment normalises this reduction (n = 15 neurons from N = 5 for sham and n = 12 neurons from N = 4 MI mice). **(e)** Lum/Iva attenuated MI-induced dendrite spine density reduction in pyramidal CA1 neurons of the hippocampus (n = 24 analysed branches from N = 4 mice). **(f)** MI attenuates hippocampal-dependent retention of object familiarity in a non-spatial novel object recognition task; Lum/Iva treatment reverses this deficit (N = 5–9; 1 mouse from MI + vehicle and 2 mice from MI + Lum/Iva groups were excluded due to total exploration times below 20 s). ∗ denotes p < 0.05 for unpaired comparisons to sham; + denotes p < 0.05 for an unpaired comparison between MI and Lum/Iva. Panels c, d, and f are presented as mean ± SEM and are compared with parametric statistical tests (ANOVA + Tukey's). Panel e is presented as median ± interquartile range and is compared with a non-parametric statistical test (Kruskal–Wallis + Dunn's).

In order to assess the therapeutic time window post–MI, we delayed treatment until 22 weeks post–MI. Neuronal deficits are clearly more severe at this time point, as evidenced by the profound reduction in dendrite intersections (Fig. 6a–c). Despite the heightened severity of the neuronal phenotype (i.e., significantly reduced number of intersections measured at 50 μm from the soma), 2 weeks of lumacaftor/ivacaftor combination therapy attenuates the MI-induced reduction in dendrite intersections (Fig. 6c, p = 0.037; ANOVA + Tukey's), normalises hippocampal dendrite lengths (Fig. 6d, p = 0.012; ANOVA + Tukey's), spine density (Fig. 6e, p = 0.004; Kruskal–Wallis + Dunn's), and long-term memory (Fig. 6f, p = 0.008; ANOVA + Tukey's). Cardiac function parameters were not affected by the treatment irrespective of type and time point of administration post–MI (Supplemental Table S1).

## Discussion

This investigation demonstrates that CFTR therapeutics restore impaired hippocampal function in an experimental model of MI. Mechanistically, our histological and *in vitro* measurements associate the activation of inflammatory mechanisms with the down-regulation of neuronal CFTR expression and the deterioration of hippocampal neuron structure in regions responsible for non-spatial memory consolidation and signal integration from the entorhinal cortex. CFTR expression plays an important neuroprotective role and thus, therapeutically restoring CFTR expression normalises the MI-induced neuronal dysfunction. This intervention displays a high degree of efficacy and appears to possess a large therapeutic time window post–MI.

There is no doubt that the immune system is activated following an MI, resulting in the elevation of pro-inflammatory cytokines within the systemic circulation.^52^^,^^53^ Indeed, our previous work demonstrating that the anti-inflammatory medication etanercept improves cognitive function following experimental MI^26^ is a strong indication that inflammation is a key driver of the neurological pathology. It is not known whether pro-inflammatory cytokines within the systemic circulation cross into the brain; however, the fact that systemically applied etanercept confers neurological benefit^26^ suggests that circulating inflammatory mediators may initiate inflammatory processes within the brain post–MI.

Microglia, the brain's primary immune cells, sense infectious invaders and stress stimuli^54^: once activated in response to these stimuli, microglia undergo a substantive morphological transformation and release pro-inflammatory cytokines. Using a qualitative morphology analysis, we confirm that microglial activation in the hippocampus is higher following MI, relative to sham-operated controls. These results complement previously published reports that describe microglia activation in the hippocampus and para-hippocampal cortex of human patients with heart disease^55^ and in the CA1 region in rodent models of heart injury.^56^^,^^57^ Chronic microglial activation is likely to result in neuropathy,^58^, ^59^, ^60^ but not all brain regions are necessarily affected similarly. For example, some studies have shown that heart injury increases pro-inflammatory cytokine levels in the hypothalamic paraventricular nucleus (PVN).^21^ Although these regions are thought to be sensitive to neuroinflammation,^22^ neuronal death in PVN and other brain nuclei was relatively sparse. This contrasts our study and others demonstrating altered neuronal structure, a reduction of synaptic protein expression^26^ and down-regulation of genes related to synaptic plasticity in different cortical and hippocampal areas.^61^

Not surprisingly, we and others show that the portfolio of cytokines released by activated microglia is substantive and includes several key entities, including IL-1β, IL-8, IL-33, chemokine (C-C motif) ligand 20 (CCL20), and IL-6.^62^, ^63^, ^64^, ^65^ These key cytokines are implicated in several neurodegenerative diseases,^66^, ^67^, ^68^, ^69^, ^70^, ^71^ with IL-8^72^ and IL-1β^73^ reported to have direct neurotoxic effects and IL-33^74^ associated with long-term memory impairment. Our *in vitro* studies show that cytokine mediators released by activated microglia down-regulate neuronal CFTR expression. The precise role of neuronal CFTR is not well defined, however it is believed to regulate synaptic inhibition and thus, hyperexcitability.^75^^,^^76^ Our observation that CFTR activity inhibition reduces PSD-95 expression, the most abundant scaffolding protein involved in the stabilisation of synapses,^77^ is consistent with this proposed function. The tight relationship between neuronal CFTR expression/activity and PSD-95 expression suggests that CFTR plays an important role in synaptic integrity, with broad implications for the maintenance of neuronal networks. In addition, reduced CFTR expression increases neuronal vulnerability to injury: we and others^78^ demonstrate that CFTR plays a key role in mitigating neuronal apoptosis following oxidative stress injury. Given that inflammation is often accompanied by oxidative stress, the reduction in CFTR expression may negatively impact neuronal health on multiple levels.

If CFTR plays a significant role in neuronal health, a neuronal phenotype should be evident in cystic fibrosis patients. Indeed, a recent landmark study shows that cystic fibrosis patients with mutations that impact CFTR production, trafficking, and/or gating defects score lower on cognitive assessments and display subtle brain tissue alterations.^79^ The lower cognitive scores observed in cystic fibrosis patients, primarily due to visual-spatial and executive impairments,^79^ align with our previous observations of cortical memory deficits in mice following MI. Intriguingly, neurodegeneration in dementia patients often originates in the entorhinal cortex (a region of the cerebral cortex that serves as the main cortical input to the hippocampus via the e.g., DG and CA1 regions) and then migrates into the hippocampus and finally the isocortex.^80^ It is not known whether a similar progression occurs in patients with MI, but it would not be surprising, given the high connectivity between hippocampus and prefrontal cortices.

Presently, CFTR medications are only used to treat cystic fibrosis; however, the revelation that pathological processes can drive “acquired” CFTR dysfunction has spurred interest in using CFTR medications outside of the cystic fibrosis patient population.^25^^,^^81^^,^^82^ Yet, the opportunities to clinically assess CFTR corrector compounds, such as lumacaftor, are severely limited, because they are not available as monotherapies. Thus, it was imperative to test an available CFTR therapeutic for efficacy in our experimental setting. The lumacaftor/ivacaftor combination medication Orkambi was an obvious choice, as it contains the effective lumacaftor ingredient. The addition of ivacaftor, a CFTR channel potentiator, would be expected to enhance CFTR activity beyond that of lumacaftor alone. Given that lumacaftor monotherapy normalised all assessment parameters, one might expect that the combination therapy would behave similarly. Demonstrating that the addition of ivacaftor is not counterproductive now opens a more straightforward path for proof-of-principle translational studies in human subjects.

Neurodegeneration is frequently portrayed as progressive and irreversible.^83^ Although the course of neurodegeneration post–MI has not been characterised, recent evidence suggests that interventions such as lifestyle changes, cardiac rehabilitation, and angiotensin-converting enzyme inhibitors can halt cognitive decline and possibly improve cognitive function.^84^, ^85^, ^86^ Since clinical HF therapy trials generally exclude patients with cognitive impairment or dementia, data pertinent to the treatment for cognitive impairment in HF patients is limited. Although early detection and treatment provides the best opportunity to limit adverse effects of MI on the brain, our data suggest that the therapeutic time window is large and that CFTR therapeutics may confer benefit even when initiated long after the MI has occurred.

As limitations, the mouse MI model utilised in the present study is able to generate clear conclusions with relatively small cohort sizes, because the animal subjects possess low genetic variation and the experimental conditions were tightly controlled. In the clinical setting, the heart failure patient population is genetically heterogenous, presents with multiple co-morbidities and are influenced by environmental factors outside of their control. The translational application of our study, therefore, requires clinical studies that integrate and assess these complexities. Although our findings indicate that inflammation-mediated neuronal CFTR reductions increase neuronal vulnerability to injury, it is important to acknowledge that non-neuronal CFTR actions may also contribute. We have previously reported that cerebrovascular CFTR expression is down-regulated post–MI, with concomitant augmentation of posterior cerebral artery tone, reduction in cerebral blood flow and impairment of cortex-dependent neurological function. We therapeutically restored CFTR expression with an experimental lumacaftor analogue,^87^ which improved all tested parameters, including perfusion.^25^ The lack of hippocampal perfusion deficits in our MI model suggests that lumacaftor improves compromised neurological function in the hippocampus by a blood flow-independent mechanism. Thus, CFTR therapeutics may have the dual action of improving perfusion in certain brain regions and increasing neuronal resilience in the post–MI setting.

A second aspect to consider is that CFTR is expressed in many organs and cells, including the lung, heart and immune system. We have previously shown that CFTR is down-regulated in the heart and lung post–MI,^25^, ^43^ as well as immune cells in the lung.^88^ With regard to the latter, alterations of immune cell-specific CFTR expression contribute to augmented lung inflammation during heart failure, thereby promoting adverse pulmonary vascular remodelling.^88^ Cardiac CFTR may play an important role in protection against acidosis/ischaemia.^89^ Thus, CFTR therapeutics potentially have system-wide effects that would not be captured by the present study's assessments. In the present study, CFTR modulator therapy lacks effects on EF, stroke volume and end-systolic/-diastolic volumes when administered long after the mature infarct scar has formed (i.e., 10 weeks post–MI): it would be intriguing to determine whether effects would be observed if the intervention would be initiated immediately following MI. Likewise, it is not known whether other CFTR-expressing organs, such as the intestine, liver, pancreas, and kidney, are affected by the systemic delivery of CFTR therapeutics. If CFTR is broadly down-regulated post–MI, CFTR therapy would likely benefit multiple organ systems. If CFTR is not broadly down-regulated, then additional research will be needed to assess the consequences of heightened expression in response to CFTR modulator therapy. Since CFTR therapeutics have been tested on healthy volunteers without serious consequences,^90^ the likelihood that this will be an issue seems remote.

Finally, the Orkambi® product data sheet indicates that a small proportion of patients (∼2%) experience a treatment-related increase in blood pressure and thus, periodic monitoring is recommended. While this adverse event is not likely to be a serious issue in the target cystic fibrosis patient population, it would be a significant concern for those with heart injury. Translational studies, therefore, would need to comprehensively assess whether the treatment alters hemodynamic parameters in patients with compromised cardiac function.

In summary, our observations indicate that microglia contribute to MI-associated neuroinflammation in the hippocampus with negative consequences for neuronal CFTR expression and hence, neuronal structure and long-term memory performance. Specifically, CFTR correctors normalise CFTR expression on hippocampal neurons and improve neuronal network alterations and memory dysfunction with a wide therapeutic time window. These findings may open the door for applying already clinically available drugs in patients with HF-associated cognitive impairment.

## Contributors

Conceptualisation, A.M.; methodology, L.V., F.E.U., C.S., J.F.D., S.R., L.U., and A.M.; validation, L.V., D.L., and A.M.; formal analysis, L.V., D.L., F.E.U., S.S., S.V., and A.M.; data curation, L.V., D.L., F.E.U., S.S., S.V., S.R., and A.M.; verified the underlying data, L.V., D.L., F.E.U., S.S., S.V., and A.M.; writing - original draft preparation, L.V., D.L., and A.M.; writing – conceptual review and editing, L.V., D.L., J.M.N.D., and A.M.; writing - review all authors; visualisation, L.V., D.L., and A.M.; supervision, A.M.; project administration, A.M.; funding acquisition, A.M., S.-S.B., L.U., and K.C.; decision to submit the manuscript, L.V., D.L., and A.M. All authors have read and agreed to the published version of the manuscript.

## Availability of data and materials

All data generated or analysed during this study are included in this published article [and its supplementary information files].

## Declaration of interests

D.L. is a consultant for Qanatpharma AG and Aphaia Pharma AG. S.-S.B. is an executive board member of Qanatpharma and Aphaia Pharma. Neither Qanatpharma nor Aphaia Pharma had any financial or intellectual involvement in this article. All other authors declare no competing interests.
