# Supplementary material for: Restoring myocardial infarction-induced long-term memory impairment by targeting the cystic fibrosis transmembrane regulator
Source: eBioMedicine. 2022 Nov 30;86:104384. doi: 10.1016/j.ebiom.2022.104384 (PMC9718964; doi:10.1016/j.ebiom.2022.104384)
Supplement: Supplemental Figure S1–S6 and Table S1–S5 [file mmc1.docx]

**Supplemental Material**

**Restoring myocardial infarction-induced long-term memory impairment by targeting the cystic fibrosis transmembrane regulator**

**Short running title:** Targeting CFTR improves MI-induced memory dysfunction

Lotte Vanherle, MSc^1,2^; Darcy Lidington, PhD^3^; Franziska E. Uhl, PhD^1,2^; Saskia Steiner, MSc^1,2^; Stefania Vassallo, MSc^1,2^; Cecilia Skoug, MSc^1,2^; Joao Duarte, PhD^1,2^; Sangeetha Ramu, MSc^1^; Lena Uller, PhD^1^; Jean-François Desjardins, MSc^4^; Kim A Connelly, MBBS^4^; Steffen-Sebastian Bolz, MD^3^ & Anja Meissner PhD^1,2,5,6^

^1^ Department of Experimental Medical Science, Lund University, Lund, Sweden.

^2^ Wallenberg Centre for Molecular Medicine, Lund University, Lund, Sweden.

^3^ Department of Physiology, University of Toronto, Toronto, Canada.

^4^ Keenan Research Centre for Biomedical Science, St. Michael’s Hospital; Toronto, Ontario, Canada.

^5^ Department of Physiology, Institute of Theoretical Medicine, Medical Faculty, University of Augsburg, Augsburg, Germany.

^6^ German Centre for Neurodegenerative Diseases, Bonn, Germany.

**Methods**

*Multiplex Elisa*

Using a Luminex immunoassay (XL Cytokine Discovery Premixed Kit, FCSTM18-15, R&D Systems, Abingdon, UK) the presence of proteins TNF-α, IL-1β, IL-12 p70, IL-8, IL-6, IL-4, IL-33, IL-13, IL-10, IFN-γ, IFN-β, GM-CSF, CCL5, CCL20 and CCL11 was measured in cell-free supernatant as per manufacturer´s instructions. Data was acquired on a Luminex MAGPIX instrument (R&D Systems, Abingdon, UK) according to the manufacturer’s instructions.

**Figures**

**
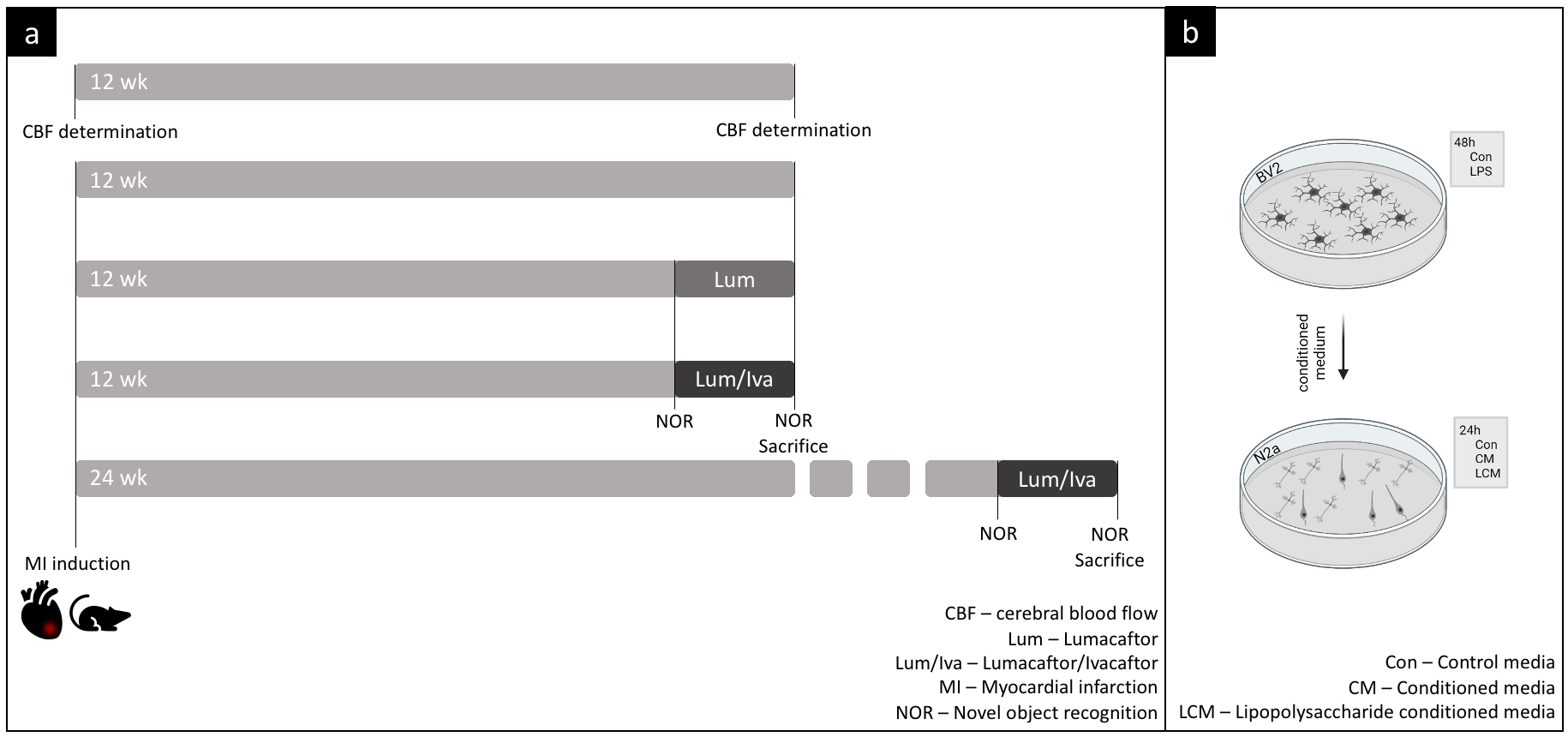
**

**Supplemental Figure 1: Overview of the experimental setup**

Panel **a** outlines the experimental approach of the *in vivo* experiments, including myocardial infarction (MI) induced by left anterior descending coronary artery ligation and behavioral testing (NOR). Treatment regimens (Lum (3 mg/kg) or Lum/Iva (3 mg/kg / 1·875 mg/kg), daily i.p. injection for 2 weeks) were started either 10- or 22-weeks post-MI. Panel **b** represents the *in vitro* experimentation. Conditioned microglia media was generated by treating BV2 microglial cells with lipopolysaccharide (100ng/ml; LCM) or vehicle control (conditioned media; CM). Subsequently, N2a neuronal cells were incubated with CM, LCM or in control media (Con) in presence or absence of Lumacaftor (10µM; Lum). Created with BioRender.

**Supplemental Figure 2: Cerebral blood flow in the hippocampus following myocardial infarction**

Using magnetic resonance imaging-based arterial spin labelling, hippocampal perfusion was measured prior to and 12 weeks following myocardial infarction (MI). Data were statistically compared with Wilcoxon matched pairs test.

**
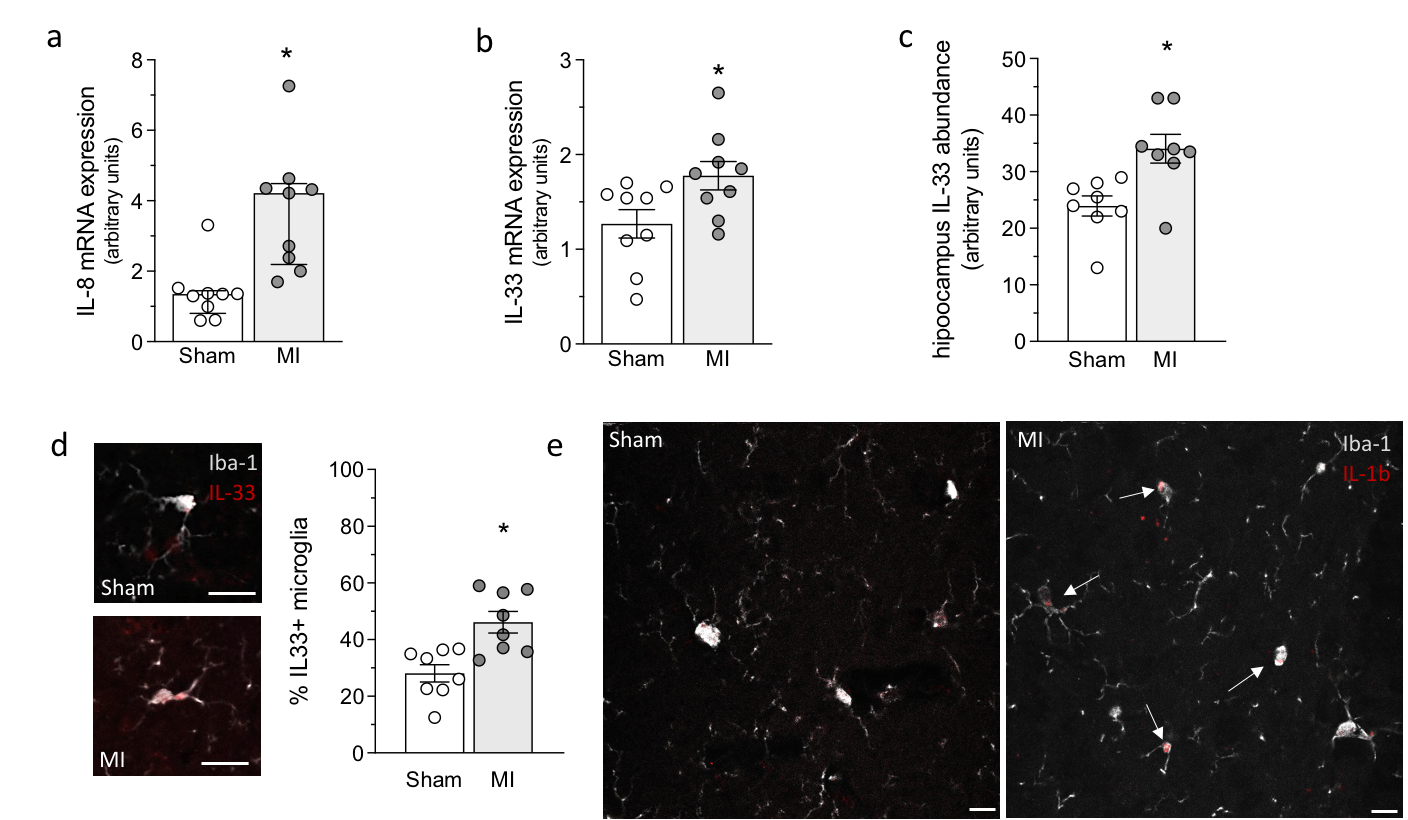
**

**Supplemental Figure 3: Cytokine expression in the hippocampus following myocardial infarction**

Myocardial infarction (MI) increases **(a)** interleukin-8 homologue (CXCL1; N=9) and **(b)** interleukin-33 (IL-33; N=9) mRNA expression in the hippocampus. **(c)** Overall hippocampal IL33 protein abundance is higher in MI mice compared to sham controls. Hippocampal microglia in the cornu ammonis and dentate gyrus region display greater cytokine positivity for **(d)** IL-33 and **(e)** IL-1β * denotes P<0.05 for unpaired comparisons. *Panel A* is presented as median ± interquartile range and is compared with a Mann-Whitney test. *Panels b, c* and *d*are presented as mean ± SEM and is compared with a Student’s t‑test. Bar over micrographs is 20 µm in *Panels d and e.*

a b

c d

**Supplemental Figure 4: Lipopolysaccharide alters activation markers in cultured BV2 microglial cells**

BV2 microglial cells were treated with vehicle (control; Con) or lipopolysaccharide (LPS; 100ng/ml) for 48 hours. LPS treatment upregulated **(a)** inducible nitric oxide synthase (iNOS), **(b)** tumour necrosis factor (TNF) and **(c)** interleukin-1β (IL-1β) mRNA expression and down-regulated **(d)** arginase-1 (Arg-1) mRNA expression. For all panels, n=9 from 3 independent experiments. * denotes P<0.05 for unpaired comparisons.  *Panels a, b and d* are presented as medians ± interquartile range and are compared with a Mann-Whitney test. *Panel c*is presented as mean ± SEM and is compared with a Welch’s‑corrected t-test.

**Supplemental Figure 5: Lipopolysaccharide does not alter CFTR protein expression in cultured N2a neuronal cells**

N2a neuronal cells were treated with vehicle (Control; Con) or lipopolysaccharide (LPS; 100ng/ml) for 48 hours. LPS treatment had no effect on cystic fibrosis transmembrane conductance regulator (CFTR; n=8 from 4 independent experiments) protein expression. Data are presented as medians ± interquartile range and are compared with a Mann-Whitney test.

**

**Supplemental Figure 6: Conditioned medium cytokine profile**

BV2 microglial cells were treated with vehicle (conditioned medium; CM) or 100ng/ml lipopolysaccharide (LPS-conditioned medium; LCM) for 48 hours. Cytokine levels were measured using a Luminex immunoassay. Targets included: tumour necrosis factor (TNF) alpha, interleukin-1β (IL-1b), interleukin-12 (IL-12) p70, interleukin-8 (IL-8), interleukin-6 (IL-6), interleukin-4 (IL-4), interleukin-33 (IL-33), interleukin-10 (IL-10), interferon (INF) gamma, granulocyte-macrophage colony-stimulating factor (GM-CSF), chemokine (C-C motif) ligand 5 (CCL5), chemokine (C-C motif) ligand 20 (CCL20), chemokine (C-C motif) ligand 11 (CCL11).

**Tables**

**Supplemental Table 1: Cardiac parameters measured by magnetic resonance imaging and echocardiography**

Cardiac parameters assessed at **(a)** 12-weeks post-surgery, **(b)**12-weeks post-surgery including 2 weeks of Lum treatment, **(c)** 12-weeks post-MI, including 2 weeks of Lum/Iva treatment, and **(d)** 24-weeks post-MI including 2 weeks of Lum/Iva treatment. Data are presented as mean ± SEM or median ± interquartile range (IQR) when highlighted in blue. * denotes P < 0.05 after One Way ANOVA or Kruskal Wallis with Bonferroni or Dunn’s post hoc testing. *BPM – beats per minute, CO – cardiac output, EDV – end diastolic volume, EF – ejection fraction, ESV – end systolic volume, HR – heart rate, Iva – Ivacaftor, Lum – Lumacaftor, MI -myocardial infarction, SV – stroke volume*

| **Primary antibody** | **Host** | **Supplier** | **Catalog #** | **RRID** | **Dilution** |
| --- | --- | --- | --- | --- | --- |
| ASC/TMS1 | Mouse | Proteintech | 67494-1-IG | AB_2882718 | 1:200 |
| CD68 | Rat | Nordic Biosite | MCA1957 | AB_322219 | 1:500 |
| CFTR | Mouse | Invitrogen | MA1-935 | AB_2081230 | 1:250 |
| Iba-1 | Rabbit | Wako | 019-19741 | AB_839504 | 1:1000 |
| IL-1β | Mouse | Santa Cruz | sc-52012 | AB_629741 | 1:100 |
| IL-33 | Goat | R&D systems | AF3626 | AB_884269 | 1:250 |
| MAP-2 | Rabbit | Abcam | ab32454 | AB_776174 | 1:500 |
| **Secondary antibody** | **Host** | **Supplier** | **Catalog #** | **RRID** | **Dilution** |
| Alexa Fluor donkey anti-Goat 594 | Donkey | Invitrogen | A-11058 | AB_142540 | 1:500 |
| Alexa Fluor goat anti-Rabbit 488 | Goat | Invitrogen | A-11034 | AB_2576217 | 1:500 |
| Alexa Fluor goat anti-Mouse 594 | Goat | Invitrogen | A-11032 | AB_2534091 | 1:500 |
| Alexa Fluor goat anti-Rat 594 | Goat | Biolegend | 405422 | AB_2563301 | 1:500 |

**Supplemental Table 2: Primary and secondary antibodies used for Immunofluorescence experiments.**

Abbreviations: *ASC – apoptosis-associated speck-like protein, CD – cluster of differentiation,* *CFTR – cystic fibrosis transmembrane regulator**, Iba-1 – ionised calcium-binding adapter molecule-1, IL – interleukin, MAP-2 –* *microtubule-associated protein 2*

| **Primary antibody** | **Host** | **Supplier** | **Catalog #** | **RRID** | **Dilution** |
| --- | --- | --- | --- | --- | --- |
| b Actin | Mouse | Sigma Aldrich | A1978 | AB_476692 | 1:3000 |
| b Tubulin | Mouse | Sigma Aldrich | T4026 | AB_477577 | 1:5000 |
| CFTR | Mouse | Invitrogen | MA1-935 | AB_2081230 | 1:1000 |
| IL1R | Rabbit | Invitrogen | GTX108702 [N3C3] | AB_1950579 | 1:1000 |
| **Secondary antibody** | **Host** | **Supplier** | **Catalog #** | **RRID** | **Dilution** |
| HRP anti-mouse | Goat | GeneTex | GTX213111-01 | AB_10618076 | 1:10.000 |
| HRP anti- rabbit | Goat | GeneTex | GTX213110-01 | AB_10618573 | 1:10.000 |

**Supplemental Table 3: Primary and secondary antibodies used for western blotting.**

Abbreviations: *CFTR – cystic fibrosis transmembrane regulator, HRP – horseradish peroxidase, IL1R – interleukin-1 receptor*

**Supplemental Table 4:** **Single comparison statistics.**

Non-highlighted data sets are presented as means ± SEM; blue-highlighted data sets are medians ± interquartile range and were subjected to non‑parametric statistical analyses and. Column *“n”* refers to the number of measures; column *“N”* refers to the number of mice. Significant differences (p values <0.05) are highlighted in red. *Abbreviations: Arg-1 – Arginase 1,* *ASC - apoptosis-associated speck-like protein containing a caspase-activation and recruitment domain*, *CD – cluster of differentiation, CFTR – cystic fibrosis transmembrane regulator, Con – control, IL – interleukin,* *IL-1R – interleukin 1 receptor, iNOS – inducible nitric oxide synthase,* LPS – lipopolysaccharide, *MFI – median fluorescence intensity, MI – myocardial infarction, PSD-95 – post synaptic density 95, TNF – tumour necrosis factor*

**Supplemental Table 5:** **Multiple comparison statistics.**

Non-highlighted data sets are presented as means ± SEM; blue-highlighted data sets are medians ± interquartile range and were subjected to non‑parametric statistical analyses and. Column *“n”* refers to the number of measures; column *“N”* refers to the number of mice. Significant differences (p values <0.05) are highlighted in red. *Abbreviations:* *CFTR – cystic fibrosis transmembrane conductance regulator, CM – conditioned medium, Con – control, LCM – lipopolysaccharide conditioned medium, MFI – median fluorescence intensity, MI – myocardial infarction, PSD-95 – post synaptic density 95*

**References**

1. Hoefer J, Azam MA, Kroetsch JT, Leong-Poi H, Momen MA, Voigtlaender-Bolz J, et al. Sphingosine-1-phosphate-dependent activation of p38 MAPK maintains elevated peripheral resistance in heart failure through increased myogenic vasoconstriction. *Circ Res.* 2010;107(7):923-33.

2. Yang J, Noyan-Ashraf MH, Meissner A, Voigtlaender-Bolz J, Kroetsch JT, Foltz W, et al. Proximal cerebral arteries develop myogenic responsiveness in heart failure via tumor necrosis factor-alpha-dependent activation of sphingosine-1-phosphate signaling. *Circulation.* 2012;126(2):196-206.

3. Meissner A, Yang J, Kroetsch JT, Sauve M, Dax H, Momen A, et al. Tumor necrosis factor-alpha-mediated downregulation of the cystic fibrosis transmembrane conductance regulator drives pathological sphingosine-1-phosphate signaling in a mouse model of heart failure. *Circulation.* 2012;125(22):2739-50.

4. Uhl FE, Vanherle L, Matthes F, and Meissner A. Therapeutic CFTR Correction Normalizes Systemic and Lung-Specific S1P Level Alterations Associated with Heart Failure. *Int J Mol Sci.* 2022;23(2).

5. Heiberg E, Sjogren J, Ugander M, Carlsson M, Engblom H, and Arheden H. Design and validation of Segment--freely available software for cardiovascular image analysis. *BMC Med Imaging.* 2010;10:1.
